# *In vitro* and *in vivo* antifungal activity of *Allium hirtifolium* and *Allium* *sativum*

**Published:** 2018

**Authors:** Alireza Diba, Fahimeh Alizadeh

**Affiliations:** *Department of Microbiology, Yasooj Branch, Islamic Azad University, Yasooj, Iran*

**Keywords:** Allium hirtifolium, Allium sativum, Candida tropicalis, Systemic candidiasis

## Abstract

**Objective:**

One of the major clinical problems is the resistance of *Candida* species towards most of antifungals. The search for new antifungal drug from plants have helped to overcome this problem. This study evaluated the effects of *Allium hirtifolium* and *Allium sativum* extracts on *Candida tropicalis* both *in vitro* and in a mouse model of systemic candidiasis.

**Materials and Methods:**

In this study, clinical isolates of *C*. *tropicalis* were isolated and identified from immunocompromised patients with recurrent candidiasis*.* Antifungal susceptibilities assessment and time kill study of aqueous and ethanolic extracts of *A. hirtifolium* and *A. sativum* extracts were done against *C*. *tropicalis*. The *in vivo* activity of aqueous extracts of *A. hirtifolium* and *A. sativum* were evaluated in a mouse model of systemic candidiasis caused by* C*. *tropicalis* through estimating the host survival time, fungal burden and histopathologic analyses.

**Results:**

The aqueous and ethanolic extracts of *A. hirtifolium* and *A. sativum *exhibited significant antifungal activity against *C*. *tropicalis*. In time kill study, *A. hirtifolium* and *A. sativum* extracts exhibited significant effects against *C*. *tropicalis *(p<0.05). Treatment of BALB/c mice that were systemically infected with *C. tropicalis*, showed that treatments with *A. hirtifolium* and *A. sativum *(at 1 mg/kg/day) were slightly less efficacious than that of fluconazole in terms of the fungal burden reduction and host survival time, it was still effective against *C. tropicalis*.

**Conclusion:**

These findings demonstrate the anticandidal properties of *A. hirtifolium* and *A. sativum* extracts *in vitro* and *in vivo* and suggest their potential to be used as an adjuvant therapy in the management of *Candida* infections.

## Introduction

*Candida tropicalis* has been considered the most prevalent candidiasis-causing yeast of the* non-albicans Candida* group (Khorsand et al., 2015 ▶; Alizadeh et al., 2017a ▶; Kord et al., 2017 ▶). Candidiasis has become a major nosocomial cause of morbidity and mortality in immunocompromised patients. Candidiasis is a diverse group of topical and systemic infections (Kothavade et al., 2010 ▶; Sardi et al., 2013 ▶). Systemic candidiasis, particularly candidemia affects over 120,000 patients annually and it had a high overall mortality rate in recent years (Zarif et al., 2010 ▶; Antinori et al., 2016 ▶).

Azole antifungals such as fluconazole and ketoconazole are being extensively used for treatment of systemic candidiasis and this extensive use has led to the rapid development of drug resistance among *Candida* species (Odds et al., 2003 ▶; Khodavandi et al., 2011a ▶). Therefore, there is an urgent need for discovery of new antimicrobial agents. Among the potential sources of new agents, plants are regarded as sources of medicinally bioactive compounds. Traditionally, *Allium hirtifolium *and *Allium sativum* have been used as antimicrobial agents. Because of their low toxicity, there is growing interest in using *A. hirtifolium* and *A. sativum* as a source of bioactive phytochemicals with antimicrobial properties against *Candida *species (Shuford et al., 2005 ▶; Katiraee et al., 2008 ▶; Palmeira-de-Oliveira et al., 2013 ▶; Khodavandi et al., 2014 ▶; Sadri et al., 2016 ▶). *In vitro* antifungal activities of *A. hirtifolium* and *A. sativum* against *C. tropicalis* were previously evaluated (Khodavandi et al., 2014 ▶; Mendoza-Juache et al., 2017 ▶); but, there has been very little data published concerning the efficacy of *A. hirtifolium* and *A. sativum* in an animal model. Therefore, in this study, the efficacy of *A. hirtifolium* and *A. sativum *was assessed by examining the *in vivo* activity of *A. hirtifolium* and *A. sativum *extracts against *C.* *tropicalis* in a mouse model of systemic infection. 

## Materials and Methods

**Patients and samples**

In this study, swab samples of vagina, mouth and skin surface of 185 immuno-compromised patients with recurrent vaginal, oral and cutaneous candidiasis, respectively were collected during a period of 14 months from August 2016 to September 2017. All patients were Iranian patients who were admitted to Shahid Beheshti Hospital affiliated to Yasooj University of Medical Sciences. Written informed consent was obtained from patients for the use of the samples in research. Clinical samples were transported to the Microbiology Laboratory, Department of Microbiology, Yasooj Branch, Islamic Azad University, Yasooj, Iran. *C.* *tropicalis* were evaluated in terms of morphological, biochemical, and molecular characteristics such as macromorphology on Sabouraud Dextrose Agar (SDA; Difco Laboratories, US), colony color on CHROMagar *Candida* medium (CHROMagar Company, France), germ tube formation, carbohydrate and nitrate assimilations, carbohydrate fermentation, urease test, and PCR amplification with universal fungal primers (ITS1 and ITS4) and DNA sequencing. The reference strain *C.* *tropicalis* ATCC 750 was used in the present study (Alizadeh et al., 2017b ▶). This study was approved by Research Ethics Committee of our institute (Ethics No. 1254039) (The study was performed according to the ethical guidelines of the 2008 Declaration of Helsinki).

**Drug, plants and animals**

Fluconazole was purchased from Sigma-Aldrich Chemicals Co. (St. Louis, MO, USA). *A. hirtifolium* and *A. sativum *were harvested in Yasooj, Iran in June 2017. Plant materials were transported to the laboratory immediately and voucher specimens were verified by Zardband Pharmaceuticals Company, Yasooj. Plants’ identity was confirmed by Dr. Azizollah Jafari Koukhdan. Female BALB/c mice (4-6 weeks old) were obtained from the Animal Breeding Stock Facility of Pasteur Institute of Iran, Karaj, Iran. 

**Plants extracts preparation**

The fresh bulbs of *A. hirtifolium* and *A. sativum *were immediately cleaned and further processed as previously described (Sadri et al., 2016 ▶), with slight modifications. Plants were washed with sterile distilled water, sliced and dried in the oven at 45°C for at least 2 days. The dried samples were blended to a fine powder and soaked in sterile distilled water or ethyl alcohol (80%, BDH, Poole, UK), at a ratio of 1: 4 (powder: solvent). The plant extracts underwent a sequential extraction using Soxhlet. The aqueous and ethanolic extracts were filter-sterilized (0.22-μm Durapore, Millipore).

**Susceptibility testing**

The disk diffusion susceptibility test was done using the Clinical and Laboratory Standards Institute (CLSI- M44-A2) with slight modification. *C.* *tropicalis* was subcultured onto SDA and grown at 35°C for 24 hr prior to testing. Next, 100 μl of inoculum (1×10^6^ to 5×10^6^ cell/ml) was poured on SDA. The prepared discs at a final concentration of 50, 60, 70, 80, 90 and 100 mg/ml of plant extracts were placed on the agar surface. Discs containing fluconazole (Sigma-Aldrich) at the concentration of 25 μg/disk were used as positive controls. The plates were incubated at 35°C for 24 hr and observed for inhibition zone (Sadri et al., 2016 ▶).

The broth microdilution method was used according to the recommendations of the CLSI (M27- A3). Susceptibility testing was performed in 96-well round-bottom microtitration plates containing 100 µl/well of twofold dilution of different concentrations of plant extracts (0.44–112.4 mg/ml) or fluconazole (0.03125–64 μg/ml) in RPMI 1640 medium with L-glutamine (Sigma-Aldrich) buffered to pH 7.0 with 0.165 M morpholinepropanesulfonic acid. Cell suspensions were prepared (1×10^6^ to 5×10^6^ cell/ml) and adjusted to a final inoculum concentration of 0.5×10^3^ to 5×10^3^ cells/ml, which were confirmed by plating them on SDA. The 96-well plates were incubated at 35°C and minimum inhibitory concentrations (MICs) were read at 530 nm using a Stat Fax 303 Reader (Awareness Technology, Inc., USA) after 24 hr. The plant extracts MICs were defined as the lowest concentration of each tested extract that caused 50% and 90 % growth inhibition compared to control-growth (without antifungals). The minimum fungicidal concentration (MFC) was defined as the lowest plant extracts concentration that resulted in ≥99.9% reduction in the starting inoculum (Khodavandi et al., 2011a ▶; Sadri et al., 2016 ▶).

**Time-kill kinetic assay**

*C.* *tropicalis* cells at a density of 1-5×10^6^ cells/ml were treated with plant extracts at a concentration equal to the MIC. After different time periods (i.e. 0, 2, 4, 6, 8, 10, 12, 24, and 48 hr) of incubation at 35 °C, the fungicidal activity of plant extracts was measured by pour plate counting method (Khodavandi et al., 2014a). Fungistatic and fungicidal activities were defined as a reduction in the number of colony forming unit (CFU)/ml, from the starting inoculum of <99.9% and ≥99.9%, respectively (Klepser et al., 1998 ▶). 

**Treatment of mice infected with** ***C.*** ***tropicalis*** **with plant extracts**

**Survival study**

*C.* *tropicalis* ATCC 750 was used for induction of the infection. The inoculum (5×10^6^ yeast cells/ml) was adjusted to 200 µl/mouse, and viable organisms were counted by pour plate counting method. Female BALB/c mice (9 mice/group) were intravenously (i.v.) infected through tail vein for inoculation of *C.* *tropicalis*. Aqueous extracts of plants (200 µl/mouse) was administrated i.v. once daily for 5 days beginning 1 hr after post-infection at 1 mg/kg/day. Comparison was made with groups of mice receiving fluconazole (200 µl/mouse, via intraperitoneal (i.p.) injections once daily for 5 days starting 1 hr post-infection at 1 mg/kg/day or normal saline for untreated control group. The survival of animals was monitored and compared to controls for 28 days post-infection (Khodavandi et al., 2011b). 

**Tissue burden study**

 For determination of tissue burden, mice were infected and treated as described above and the tissue burden was assessed on days 2, 4, 7, 10, 14 and 28 post-infection. The kidneys were aseptically removed and homogenized in 1 ml of sterile normal saline and cultured on SDA plates as explained in the time-kill kinetic assay, and assessed by determination of fungal colonization (Khodavandi et al., 2011b ▶).

**Histopathological analyses**

 Tissues were fixed in 10% neutral buffered formalin (Merck, Germany) for at least 2 days and blocked by paraffin wax using a Shandon Automated Tissue Processor (ThermoShandon, PA, USA). The blocked tissues were cut using a Leica microtome (Leica, model RM2025, Germany) in 4-µm thickness. The sections were stained by hematoxylin and eosin (H&E) and periodic acid shiff's (PAS) and viewed under light microscopy (Leica, DMRA II, Germany; Khodavandi et al., 2011b ▶). All animal care and use were approved by the Islamic Azad University Animal Ethics Committee (95/2/14-26). 

**Statistical analysis**

Data obtained from *in vitro* inhibitory effects of plant extracts and also tissue burden studies, were examined in terms of normality. Differences among different groups were determined by one-way ANOVA. Log Rank and Tukey's tests were used for determination of survival time. A p value <0.05 was considered significant. SPSS Statistics version 24 (SPSS Inc., Chicago, IL) was used for statistical analysis. All experiments were carried out at least in triplicates.

## Results

Of patients with candidiasis from whom we obtained samples, 43% were male and 57% were female. Patients’ age ranged from 24 to 78 years old with a mean of 39 years old. In this study, 180 (97.30%) *Candida* isolates were identified. Among *Candida* isolates, 73.33% were *C*. *albicans*, 20.55% were *C. krusei,* 5.56% were *C. tropicalis* and 0.56% were *C. glabrata*. Clinical isolates of *C.* *tropicalis* were used in this study. In addition, all *C.* *tropicalis* isolates were sensitive to fluconazole and no drug resistance was seen. 

Susceptibility to aqueous and ethanolic extracts of *A. hirtifolium* and *A. sativum* are summarized in Table 1. The results revealed that aqueous extract of *A. hirtifolium* strongly inhibited the activity of *C.* *tropicalis* isolates at a concentration of 100 mg/ml*. *Table 2 shows the MICs and MFC of plant extracts against *C.* *tropicalis* isolates.

**Table 1:** Antifungal activity of *A. hirtifolium* and *A.* *sativum* (as reflect by inhibition zone (mm)) against clinical isolates of *C. tropicalis* by disk diffusion susceptibility assay at 100 mg/ml concentration after 24 hr incubation at 35°C.

**Isolates/ Extracts**	***A. hirtifolium***		***A. *** *** sativum***		**Fluconazole**
	**Aqueous** **extract**	**Ethanolic** **extract**	**Aqueous** **extract**	**Ethanolic** **extract**	**25 μg/disk**
**Ct** **ATCC**	26.00±1.79	23.00±1.87	25.00±1.99	24.00±1.27	10.70±0.12
**Ct1**	26.00±2.20	24.00±2.00	24.00±2.11	23.00±2.09	11.90±0.10
**Ct2**	25.00±1.14	25.00±1.08	24.00±1.67	24.00±1.70	11.70±0.11
**Ct3**	26.00±1.09	24.00±1.48	26.00±1.76	24.00±1.98	10.00±0.20
**Ct4**	26.00±1.99	24.00±1.02	25.00±1.29	23.00±1.70	13.00±0.20
**Ct5**	25.00±1.77	25.00±1.50	24.00±2.21	24.00±1.34	10.10±0.28
**Ct6**	26.00±2.09	23.00±2.03	26.00±2.16	23.00±1.78	11.00±0.09
**Ct7**	25.00±1.56	23.00±1.44	24.00±1.22	24.00±1.55	10.20±0.10
**Ct8**	25.00±1.89	24.00±1.44	25.00±1.33	24.00±1.57	12.00±0.21
**Ct9**	26.00±1.68	25.00±1.55	26.00±2.11	21.00±2.09	10.72±0.23
**Ct10**	25.00±1.68	25.00±1.43	24.00±1.13	24.00±1.22	11.12±0.20

Ct: *C. tropicalis.*

Data are expressed as means±standard deviation of three independent experiments.

**Table 2:** Antifungal activity of *A. hirtifolium* and *A.* *sativum* against clinical isolates of *C. tropicalis* as assessed by broth microdilution assay after 24 hr incubation at 35°C**.**

**Isolates/ Extracts**	***A. hirtifolium*** *			***A. *** *** sativum*** *			**Fluconazole** **		
**Aqueous **									
	**MIC**_50_	**MIC**_90_	**MFC**	**MIC**_50_	**MIC**_90_	**MFC**	**MIC**_50_	**MIC**_90_	**MFC**
**Ct** **ATCC**	6.27±0.32	11.28±1.12	12.54±0.56	11.27±0.78	21.12±1.22	24.12±0.98	0.80±0.09	2.50±0.20	3.30±0.25
**Ct1**	6.25±0.45	11.26±1.31	12.51±1.09	10.25±1.45	21.26±1.33	25.51±1.22	0.60±0.02	2.30±0.10	3.20±0.23
**Ct2**	6.80±0.66	12.24±0.89	13.60±1.11	11.11±0.98	22.24±0.98	23.60±1.33	0.60±0.10	3.30±0.20	4.40±0.32
**Ct3**	6.82±0.56	12.27±0.99	13.64±1.15	10.25±1.56	22.27±0.99	23.64±1.23	0.50±0.00	2.60±0.30	3.20±0.24
**Ct4**	6.57±0.45	11.82±0.67	13.14±0.98	10.57±1.45	21.82±0.79	25.14±1.98	0.70±0.08	3.50±0.20	4.50±0.33
**Ct5**	6.55±0.44	11.80±1.12	13.10±1.09	9.55±0.99	22.80±1.22	23.10±1.45	0.50±0.02	2.50±0.30	3.50±0.33
**Ct6**	6.36±0.29	11.44±1.18	12.72±1.45	11.23±0.99	21.22±1.22	24.14±1.55	0.60±0.10	3.60±0.10	4.40±0.33
**Ct7**	6.80±0.48	12.24±1.56	13.60±1.12	12.80±1.48	22.24±1.55	25.55±1.45	0.50±0.20	3.50±0.40	4.50±0.35
**Ct8**	6.4±0.34	11.55±1.13	12.84±0.96	10.42±0.98	21.33±1.33	24.33±1.96	0.70±0.10	2.50±0.20	3.50±0.42
**Ct9**	6.75±0.69	12.15±1.66	13.50±1.03	11.75±1.34	22.15±1.99	25.50±1.44	0.80±0.07	2.90±0.30	3.30±0.25
**Ct10**	6.57±0.68	11.82±0.99	13.14±0.87	10.57±1.11	21.21±1.99	24.33±1.87	0.50±0.08	3.50±0.10	4.20±0.15
**Ethanolic**									
	**MIC**_50_	**MIC**_90_	**MFC**	**MIC**_50_	**MIC**_90_	**MFC**	**MIC**_50_	**MIC**_90_	**MFC**
**Ct** **ATCC**	6.78±0.25	12.20±1.11	13.56±0.99	11.11±1.25	22.09±1.21	23.56±0.78	0.80±0.09	2.50±0.20	3.30±0.25
**Ct1**	6.30±0.45	11.30±1.34	12.56±1.01	12.22±1.16	21.23±1.55	23.12±1.34	0.60±0.02	2.30±0.10	3.20±0.23
**Ct2**	6.55±0.33	11.79±1.09	13.10±0.88	11.55±0.78	23.79±1.44	25.33±0.99	0.60±0.10	3.30±0.20	4.40±0.32
**Ct3**	6.90±0.56	12.42±0.97	13.80±0.98	10.90±0.89	22.42±0.99	23.44±0.77	0.50±0.00	2.60±0.30	3.20±0.24
**Ct4**	6.25±0.57	11.25±1.13	12.50±0.78	11.25±1.09	23.25±1.08	25.58±1.78	0.70±0.08	3.50±0.20	4.50±0.33
**Ct5**	6.25±0.22	11.25±0.99	12.50±0.48	11.25±0.98	23.45±1.99	25.11±1.11	0.50±0.02	2.50±0.30	3.50±0.33
**Ct6**	6.15±0.43	11.07±1.25	12.30±1.13	12.12±1.12	22.21±1.34	23.44±1.44	0.60±0.10	3.60±0.10	4.40±0.33
**Ct7**	6.25±0.14	11.25±0.58	12.50±0.98	11.25±1.14	23.25±0.99	25.50±0.88	0.50±0.20	3.50±0.40	4.50±0.35
**Ct8**	6.27±0.59	11.28±0.89	12.54±0.37	12.27±1.13	23.28±0.99	25.46±0.99	0.70±0.10	2.50±0.20	3.50±0.42
**Ct9**	6.23±0.71	11.21±0.76	12.46±0.76	11.23±0.99	22.21±0.88	25.46±0.89	0.80±0.07	2.90±0.30	3.30±0.25
**Ct10**	6.35±0.29	11.43±1.01	12.70±0.88	12.45±1.44	23.43±1.67	24.90±0.72	0.50±0.08	3.50±0.10	4.20±0.15

Ct: *C. tropicalis.*

* MIC and MFC values are expressed as mg/ml obtained from three independent experiments.

** MIC and MFC values are expressed as μg/ml obtained from three independent experiments.

**Figure 1:**
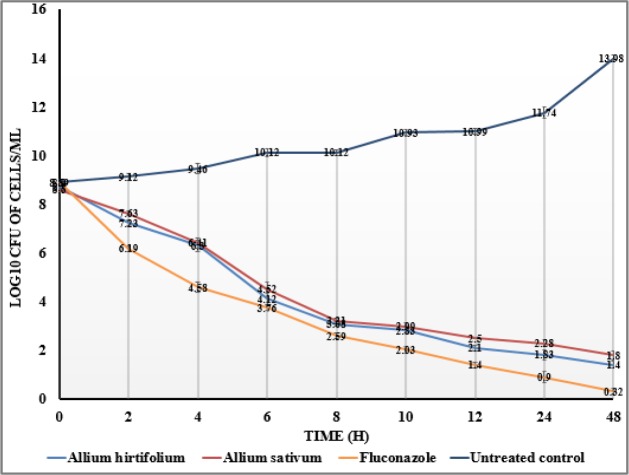
Time-kill curves of *A. hirtifolium* and *A. sativum* against *C. tropicalis *at different time points. Plant extracts were tested at a concentration equal to MIC.

Figure 1 shows the potency of aqueous extracts of *A. hirtifolium* and *A. sativum* in decreasing the cell number of* C. tropicalis* ATCC 750 after 0, 2, 4, 6, 8, 10, 12, 24, and 48 hr. The numbers of viable *C. tropicalis* cells at different time points were significantly reduced compared to untreated control (p<0.05). *C. tropicalis* treated with *A. hirtifolium* and *A. sativum* demonstrated endpoint activity (<99.9% reduction in numbers of CFU/ml from the starting inoculum) at different time points*.* The observed changes in CFU/ml for ethanolic extracts of plants against *C. tropicalis* were similar to those observed for aqueous extracts (Data not shown).

**Figure 2:**
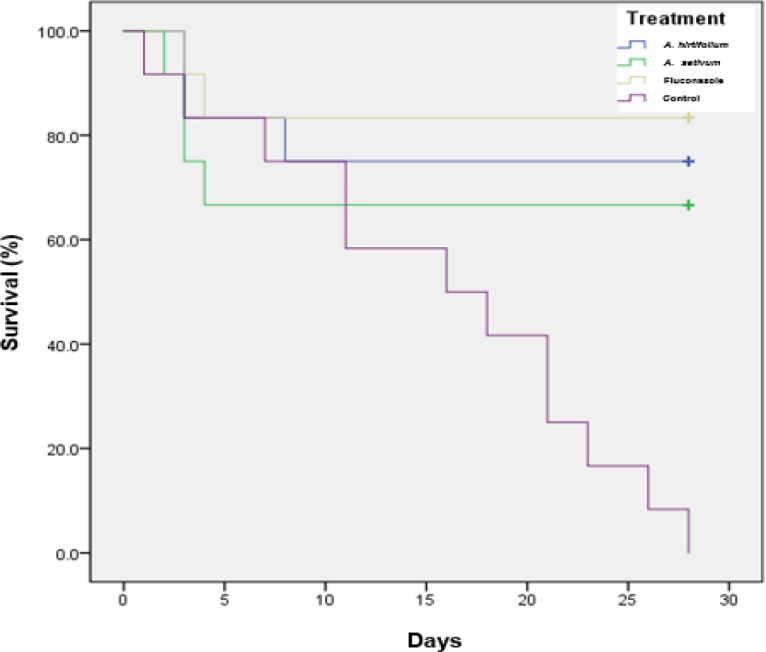
Cumulative mortality of mice infected with *C. tropicalis *treated with *A. hirtifolium,* *A. sativum* and fluconazole (p<0.05).

*In vivo* efficacy of plant extracts against *C. tropicalis* infection was evaluated in infected mice during a period of 28 days post-infection. The mean survival time of mice treated with* A. hirtifolium*, *A. sativum, *fluconazole and untreated control were 22.17±2.94, 19.67±3.40, 23.92±2.64 and 15.50±2.57 days, respectively. Moreover, based on statistical analysis of Log Rank=20.31±1.55 in this study, the mean survival time between treated and control groups indicated significant differences (p<0.05; Figure 2). 

**Table 3:** Tissue fungal loads (Log_10_ CFU/g of kidney tissue±SD) obtained from mice infected with *C. tropicalis *ATCC 750 and treated with *A. hirtifolium* and *A.* *sativum.*

**Days post-infection **	***A. hirtifolium***	***A. *** *** sativum***	**Fluconazole**	**Untreated Control**
**2**	4.19±0.61^a^	4.20±0.37^a^	4.26±0.47^ab^	4.31±0.39^b^
**4**	3.93±0.36^b^	3.95±0.23^c^	3.62±0.31^a^	4.41±0.48^d^
**7**	3.82±0.33^b^	3.84±0.24^c^	3.52±0.30^a^	5.11±0.55^d^
**14**	3.31±0.26^b^	3.29±0.53^c^	3.09±0.44^a^	5.29±0.47^d^
**28**	3.08±0.39^b^	3.10±0.36^c^	2.74±0.35^a^	6.34±0.36^d^

Results in a row with different superscripts differ significantly (p*<*0.05) as assessed by Tukey's test.

**Figure 3:**
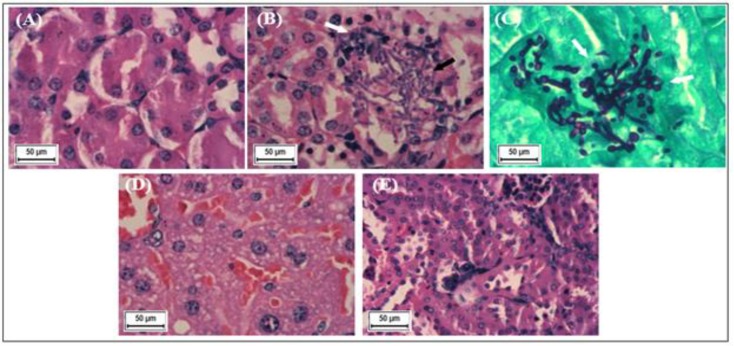
Histological structures of normal, untreated- and treated-mouse kidney sections. (A) Normal kidney section of mice showing closely-packed tubules lined by simple cuboidal epithelium. The interstitial tissue between the tubules is indistinct, and no *C. tropicalis *organisms or inflammatory cells were seen (H&E X400); (B) Positive control (without treatment) of an infected mouse kidney section, showing *Candida* yeasts and hyphae (black arrow) between the tubules, as well as a few scattered lymphocytes (white arrow). Also, the interstitial space shows moderate congestion, with dilated blood vessels (H&E X400); (C) Positive control (without treatment) of an infected mouse kidney section stained with PAS, highlighting the presence of *Candida* yeasts and hyphae (white arrows) (PAS X400). (D) Kidney section from mice treated with *A. hirtifolium* showing reduced (mild) congestion within the interstitial tissue and inflammatory cells (H&E X400); (E) Kidney section from mice treated with fluconazole, showing normal appearance of closely-packed tubules without any congestion, inflammatory cells or *Candida* organisms (H&E X400).

*A. hirtifolium* and *A. sativum* were efficient at inhibiting the growth of *C. tropicalis*, *in vivo*. The results of fungal burden determination in the kidney at different time points, indicated a significant reduction in CFU/g in the tissue (p*<*0.05) starting from day 2 post-infection. Cell viability was significantly higher in mouse kidney tissues treated with *A. hirtifolium* in comparison to those treated with* A. sativum*, 28 days post-infection (Table 3). Histopathological analysis showed the presence of *C. tropicalis* yeasts and hyphae between the tubules, as well as a few scattered lymphocytes and moderate congestion in the infected kidney 10 days post-infection. While in kidney section from mice treated with *A. hirtifolium*, mild congestion within the interstitial tissue was observed. On the other hand, kidney section from mice treated with fluconazole, showed a normal appearance of closely-packed tubules without any congestion (Figure 3). Histopathological analysis of infected kidney sections from mice treated with* A. sativum* demonstrated mild congestion within the interstitial tissue (Data not shown).

## Discussion

Plant and their active compounds have been extensively studied for antifungal activities (Shuford et al., 2005 ▶; Katiraee et al., 2008 ▶; Palmeira-de-Oliveira et al., 2013 ▶; Khodavandi et al., 2014 ▶; Sadri et al., 2016 ▶). In this study, we investigated the potency of *A. hirtifolium* and *A. sativum* against *C. tropicalis* isolated from human infections. Our findings were consistent with previous observations which demonstrated the antifungal activity of *A. hirtifolium* and *A. sativum* (Khodavandi et al., 2014 ▶; Sadri et al., 2016 ▶; Mendoza-Juache et al., 2017 ▶).

The results of antifungal activity measurement by disk diffusion susceptibility method demonstrated that zones of inhibition caused by aqueous and ethanolic extracts of* A. hirtifolium* and *A. sativum* against *C. tropicalis* of were 25.50±0.53 mm, 24.20±0.79 mm, 24.80±0.92 mm and 23.40±0.97 mm, respectively. The results for aqueous and ethanolic extracts of* A. hirtifolium* and *A. sativum* against *C. tropicalis* measurement by broth microdilution method showed MIC_90_s of 11.66±0.36 mg/ml, 11.42±0.40 mg/ml, 21.85±0.57 mg/ml and 22.85±0.80 mg/ml, respectively. This study indicated that aqueous extract of *A. hirtifolium* has stronger antifungal activity against *C. tropicalis.* The chemical composition of aqueous extracts could be responsible for the biological activities (Ghahremani-majd et al., 2012; Lanzotti et al., 2013; Mnayer et al., 2014; Szychowski et al., 2016). The time-kill kinetic curves of plant extracts tested against *C. tropicalis* indicated a significant inhibition of growth when comparing untreated controls with those treated with plant extracts and fluconazole after 2 hr of incubation. This demonstrates that *A. hirtifolium* and *A. sativum* decreased the growth of *C. tropicalis* almost as efficiently as fluconazole (p>0.05). Previous reports have demonstrated the *in vitro* antifungal activity of *A. hirtifolium* against *C. tropicalis* (Khodavandi et al., 2014 ▶; Mendoza-Juache et al., 2017 ▶).

The chemical composition and bioactive compounds of *Allium* plants have been reported especially the organosulfur compounds which include diallyl trisulfide, diallyl-dithiosulfinate (allicin), diallyl disulfide and S-allylcysteine. Additionally, *Allium* plants contain polyphenolic compounds like flavonoids (Ghahremani-majd et al., 2012 ▶; Lanzotti et al., 2013 ▶; Mnayer et al., 2014 ▶; Szychowski et al., 2016 ▶). Organosulfur compounds present in *A. hirtifolium* and *A. sativum* are the most important chemicals responsible for the biological effects of these herbs (Mikaili et al., 2013 ▶). The bioactive compounds in *A. hirtifolium* are likely to be responsible for the antifungal activities sought (Leelarungrayub et al., 2004 ▶). 

The underlying mechanism of anticandidal effect of *Allium* plants are not well understood. However, *A. sativum* have been shown to decrease the oxygen uptake, induce oxidative stress, inhibit the synthesis of lipids, proteins and nucleic acids, damage cell membrane integrity and reduce the growth of the organism (Ghannoum, 1988 ▶; Khodavandi et al., 2011b ▶; Bayan et al., 2014 ▶). Li et al., 2016 ▶ indicated that *A. sativum* could penetrate the cellular membrane of *C. albicans* as well as the membranes of organelles such as the mitochondria, resulting in organelle destruction and ultimately cell death. Diallyldisulfide caused a decrease in the activity of all antioxidant enzymes except catalase (Mikaili et al., 2013 ▶). Biological activities of *A. hirtifolium* could be due to the presence of various chemicals, mainly sulfur-containing and polyphenolic compounds (Ghahremani-majd et al., 2012 ▶; Mikaili et al., 2013 ▶). Khodavandi et al. found that *A. hirtifolium* downregulates the expression of hypha specific gene *HWP1 *which possibly explains the inhibitory effect of *A. hirtifolium* on hyphae and biofilm formation in *C. albicans *(Khodavandi et al., 2014 ▶). Sadri et al. proposed that *A. sativum*, *A. hirtifolium* and *A. cepa* extracts differentially expressed transcriptional repressor and hypha-specific genes in *C. albicans* (Sadri et al., 2016 ▶). 

The obtained results from *in vivo* experiments showed that the extracts of* A. hirtifolium* and *A. sativum* inhibited the growth of *C. tropicalis*. The results of the tissue burden studies demonstrated a significant inhibition of *C. tropicalis* growth following treatment with *A. hirtifolium, A. sativum* and fluconazole in comparison to untreated controls after 4 hr. In addition, high percentage of survival among *C. tropicalis*-infected animals, was observed. The survival study showed that *A. hirtifolium* and *A. sativum* could increase the mean survival time until day 22 and 20, respectively whereas the untreated control group showed a mean survival time of 15.5 days. The clearance rate of *C. tropicalis* from the kidneys of infected animals that received *A. hirtifolium* was comparable to those treated with fluconazole. Moreover, the clearance rate of *Candida* cells from the kidneys of infected animals that received *A. sativum* was identical to that observed following treatment with *A. hirtifolium*. This finding showed that *A. hirtifolium* decreased the growth of *C. tropicalis* with similar efficiency to fluconazole, demonstrating a comparable ability to inhibit the growth of the yeast cells. Little is known about the potential *in vivo *anticandidal activity of *Allium*. It has been reported that the organosulfur compounds from *A. sativum* target multiple pathways and cause mutagenesis inhibition, enzyme activities modulation, and inhibition of DNA adduct while affecting the intrinsic pathway of apoptotic cell death and cell cycle machinery (Omar and Al-Wabel, 2010 ▶). 

The results from *in vitro* and *in vivo* studies in the present work demonstrated that *A. hirtifolium* is slightly less efficacious than fluconazole in the treatment of candidiasis. Also, results demonstrated that* A. sativum* is slightly less efficacious than* A. hirtifolium*. These findings suggest the potential of *A. hirtifolium* and *A. sativum* extracts to be used as an adjuvant therapy against *Candida* infections. This model of treatment warrants further evaluations for possible clinical applications. 
